# On Modern Progress in Ophthalmic Medicine and Surgery

**Published:** 1895-02-16

**Authors:** Robert Brudenell Carter

**Affiliations:** Consulting Ophthalmic Surgeon to St. George's Hospital


### ON MODERN PROGRESS IN OPHTHALMIC MEDICINE AND SURGERY.

By ROBERT BRUDENELL CARTER, F.R.C.S., Consulting Ophthalmic Surgeon to St. George's Hospital.

### DISEASES OF THE CORNEA.

THE integrity of the cornea, or transparent window which forms the front of the eye, is a matter of primary necessity for the possession of good vision. The slightest cloud upon its transparency is sufficient to obscure the outlines of objects; the slightest departure from its natural curvature is sufficient to produce apparent distortion of their forms. It is liable, of course, to sustain injuries which may be more or less serious in their consequences; and it is liable to suffer from inflammations and ulcerations of various kinds and various degrees of severity.

The healthy cornea is of absolute transparency, permitting the finest details of the surface of the iris to be clearly seen through it, and it furnishes, as a convex mirror, diminished erect images of the objects from which it receives light. If a patient be seated opposite a window, images of that window will be seen reflected from his corneæ. If the corneal surface be in the least dull, as if it had been breathed upon, or if it present any other appearance than that of a highly polished and perfectly transparent convex membrane, its natural structure has undergone some change, and its efficiency as a portion of the visual apparatus must be impaired in a corresponding degree.

The exposed position of the cornea in front of the eye involves a liability to frequent injury from the impact or lodgment of a foreign body, and there are few railway travellers who have not suffered from this cause. Pieces of coal or grit may readily be projected against the eye with such force as to become partially imbedded, and incapable of removal by the action of the lids; or they may produce as much, or even more, discomfort if they pass under the upper lid, adhere to its inner surface, and scrape the corneal epithelium with every movement. I have seen, in the course of my life, a very considerable number of cases in which one or other of these conditions had remained undetected, sometimes for weeks, or even months, and had occasioned a so-called "inflammation," against which all the resources of treatment had been invoked in vain. Generally speaking, foreign bodies are conspicuous enough when they are looked for, but this is not always the case. A tiny splinter of glass may easily escape notice by its resemblance to the corneal tissue, and a perfectly black fragment, as of coal, may be invisible against the black background of the pupil. Whenever inflammation of the eye is of sudden occurrence, a foreign body should be looked for, at first under the upper lid, by everting it, and next upon the cornea, which should be examined in a good light and at different angles. In some cases it will be necessary to have recourse to what is called focal illumination, by focussing the light of a lamp upon the surface with a convex lens, while another lens is employed as a magnifier. I once discovered in this way a minute particle of white sand from a Belgian road, which was imbedded in the upper third of the cornea, and which had curtailed the Continental holiday of its unlucky bearer by sending him home for treatment. A few years ago, with timid or unsteady patients, the removal of a foreign body was often a task of considerable difficulty, in the accomplishment of which the corneal epithelium was apt to be scratched somewhat extensively. All such difficulty is now removed by the use of cocaine. A wafer containing the fiftieth of a grain of the alkaloid should be placed within the conjunctiva of the lower lid, and in ten or twelve minutes the foreign body may be picked off the surface by forceps, or tilted out from its bed by a little spud or spatula. Sometimes it will be found to have penetrated somewhat deeply, and to be situated obliquely among the corneal layers, in which case it may be necessary to cut through the overlying tissue with the point of a cataract knife before either the forceps or the spatula can be applied. Whenever the corneal wound is at all extensive it will be liable to receive septic matter from ths atmosphere, or it may have been septicised in the first instance by the foreign body itself. It should, therefore, be carefully cleansed by some aseptic wash, such as a 15 per cent. solution of Barff's boroglyceride, and the lids should be closed with a strip or two of isinglass plaster until healing is accomplished. Mere abrasions of epithelium will be repaired in a few hours, and seldom give any trouble, but even with them it is a good plan to wash the surface, and then to protect it by the instillation of a drop of castor oil, in which, if necessary, a little atropine, or cocaine, or both, may be dissolved. The oil forms an impervious film over the surface, and often affords immediate relief from discomfort.

A foreign body which not only wounds the cornea, but passes completely through its thickness, and opens the anterior chamber, is generally productive of consequences beside which those of the corneal wound itself are comparatively unimportant, and which would come naturally to be dealt with among severe injuries of the eye. It may happen, nevertheless, that division of the cornea is the sole effect of an injury, the projectile not having reached the iris or lens, and having been thrown out again by the resiliency of the wounded tissue. The line of cicatrix, in such a case, is likely to be permanently opaque, and the extent to which it will impair vision will depend upon its breadth, and upon its position with regard to the pupillary area. Both of these will be mainly determined by the character and position of the wound, and the chief duty of the surgeon will be to prevent adhesion of the iris to the cicatrix. This may be done by the instillation of atropine if the wound be central or nearly so; or by the instillation of eserine if the wound be marginal, the object in either case being to remove the edge of the pupil from contact with it. If the anterior chamber be so freely opened that the entrance of any fluid used for washing the surface of the wound is probable, it is best to employ for this purpose a sterilised solution of common salt, of ½ per cent., and, when the wound has been cleansed as far as possible, to close the lids with plaster and a wool compress, and to avoid opening them, unless compelled to do so by inflammation or swelling, until the process of cicatrisation is complete.

